# Gene structure of three kinds of vacuolar-type Na^+^/H^+^ antiporters including *TaNHX2* transcribed in bread wheat

**DOI:** 10.1590/1678-4685-GMB-2020-0207

**Published:** 2021-04-23

**Authors:** Motonori Tomita, Masakazu Yamashita, Akihiro Omichi

**Affiliations:** 1 Shizuoka University, Research Institute of Green Science and Technology, Genetics and Genome Engineering Lab, Shizuoka, Shizuoka 422-8529, Japan.; 2 Tottori University, Faculty of Agriculture, Molecular Genetics Lab, Tottori, Tottori, Japan.

**Keywords:** Wheat, vacuolar-type Na^+^/H^+^ antiporter, CapFishing, gene structure, phylogenetic relation

## Abstract

The vacuolar-type sodium/proton antiporter is considered to play an important role in withstanding salt stress by transporting sodium ions into vacuoles. In this study, the gene structures of three kinds of vacuolar-type antiporters transcribed in bread wheat under salt stress were analyzed. After spraying 0.5 M NaCl to seedlings of wheat cultivar Chinese Spring, 1,392~1,400 bp cDNA fragments were isolated by RT-PCR using primers designed from common regions in rice *OsNHX1* and *Atriplex subcordata AgNHX1*. Next, the entire structure of the genomic DNA and cDNA were determined via CapFishing-5’ Rapid Amplification of cDNA Ends (RACE), 3’RACE, and genomic PCR cloning. As a result, 3 kinds of vacuolar-type Na^+^/H^+^ antiporter genes, *TaNHXa* (genome DNA 4,255 bp, cDNA 2,414 bp, 539 a.a.), *TaNHXb* (gDNA 4,167 bp, cDNA 1,898 bp, 539 a.a.) and *TaNHXc* (gDNA 4,966 bp, cDNA 1,928 bp, 547 a.a.), were identified. They encode 12 transmembrane domains containing third domain’s amyloid binding sites (FFIYLLPP), characteristic of the vacuolar-type Na^+^/H^+^ antiporter, binding to the cell vacuolar membrane. *TaNHXa*, *b* and *c* consisting of 14 exons and 13 introns were 22~55 % longer than *A. thaliana AtNHX1* in total length*. TaNHXa* (*TaNHX2*) showed homogeneity with *OsNHX1*, while *TaNHXb* and *c* were phylogenetically independent.

## Introduction

Salinity is a global major abiotic constraint to crop production. Sodium at high millimolar levels in the cytoplasm is toxic to plants. Understanding the uptake and transport properties of salt in plants is crucial to evaluate their potential for growth in high salinity soils and as a basis for engineering varieties with increased salt tolerance. Ion transporters, representative as monovalent cation/proton antiporters, play an essential role in ion homeostasis, mainly for Na^+^, Cl^-^, and K^+^. The Na^+^/H^+^ antiporters encoded by NHX gene family are membrane proteins that catalyze the Na^+^-H^+^ exchange across vacuolar membranes (Qiu, 2012). Sodium/proton antiporters are widely distributed in living organisms, including bacteria and animals (Counillon and Pouyssegur, 2000; Chanroj et al., 2012). They serve as pumps that countertransport a single sodium ion to a single proton across a biological membrane to regulate intracellular Na^+^ concentration (Hanana et al., 2009). Sequestration of Na^+^ ions into the vacuole through the action of Na^+^/H^+^ antiporters is one mechanism that confers salt tolerance to organisms (Al-Harrasi et al., 2020). In plants, these are believed to be involved in the excretion of sodium ions entering the cell and sequestration into vacuoles, which occupy most of the cell volume under salt stress (Jia et al., 2018). Plant Na^+^/H^+^ antiporters (NHXs) are pivotal regulators of intracellular Na^+^/K^+^ and pH homeostasis, which is essential for salt stress adaptation (Ayadi et al., 2020; Li et al., 2020). The vacuolar type, in particular, is considered to play an important role in withstanding salt stress by transporting sodium ions into vacuoles (Jia *et al.*, 2018). A vacuolar sodium/proton antiporter has been cloned from several plants (Fukuda et al., 1999; Hamada et al., 2001; Wang et al., 2002; Al-Harrasi *et al.*, 2020; Ayadi *et al.*, 2020) and the genes of plasma membrane-type sodium/proton antiporters have been identified in *Arabidopsis thaliana* (Apse et al., 1999; Gaxiola et al., 1999;). *AtNHX* from *A. thaliana* and *AgNHX* from *A. subcordata* have been shown to improve salt tolerance through transgenic experiments in *A. thaliana*, Western rapeseed, and rice (Apse *et al.*, 1999; Zhang et al., 2001; Yokoi et al., 2002).

In wheat, one of the three most abundant crops in the world, several sodium/proton antiporter genes have been cloned (Brini et al., 2005; Sharma et al., 2020). From wheat, it may be possible to develop resistant plants against salt stress in semi-arid land. The development of salt-tolerant varieties that can withstand salt stress is promising. However, whole genomic exon/intron structures were not clear in wheat NHX. In this study, firstly, cDNAs from three vacuolar antiporter genes (*TaNHXa, b*, and *c*) that are transcribed in bread wheat under salt stress were isolated and their total structures were determined by CapFishing-5’ Rapid Amplification of cDNA Ends (RACE) and 3’RACE methods. Moreover, we revealed their whole genomic structures by sequencing genomic PCR products by primers designed from cDNAs.

## Material and Methods

### Cloning of Na^+^/H^+^ antiporter-like cDNA sequences

Wheat cultivar Chinese Spring grown at 25 ℃ for a 12 h day length for approximately 1 month, was sprayed with 0.5 M NaCl and the leaves were sampled from the seedlings. Total RNA was extracted from the leaves using the Acid Phenol Guanidium Chloroform method (Chomczynski and Sacchi, 2006), and 50 μg of total RNA was then treated with 10 U of DNase I (Takara Bio Inc., Kyoto, Japan). Single-stranded cDNAs were synthesized by reverse transcription reaction using 10 U AMV reverse transcriptase XL (Takara Bio Inc., Kyoto, Japan) for 30 min at 42 ℃ and 15 min at 70 ℃ by using 1 μg of total RNA as templates of 125 μM oligo(dT) primer in 40 μL solution (1 mM dNTP, 5 mM MgCl_2_. 40 U RNase Inhibitor, 1×RNA PCR buffer). To clone Na^+^/H^+^ antiporter genes expressed in bread wheat, primers were designed from common regions in *OsNHX1* of rice and *AgNHX1* of *A. subcordata*. The a forward primer, Na/H-F [5’-GGAGAATCGCTGGGTCAATGAGTCC-3’] was designed from the nucleotide sequence 425-449 bp region of *OsNHX1* and the 816-840 bp region of *AgNHX1*. Meanwhile, a reverse primer Na/H-R [5’-TCAGCGCGTCGTCGAACTTGCGC-3’] was selected from the 1,802-1,824 bp region of *OsNHX1* and the 2,235-2,257 bp region of *AgNHX1*. Primer sequences designed in this study were compiled in Table 1. By using 4 μL of the RT reaction solution as a template, 1 μM of forward and a reverse primers, 1 mM dNTPs, 50 mmol/L KCl, 10 mmol/L Tris-HCl (pH 8.8), 2.5 mM MgCl_2_, 1×LA PCR buffer Ⅱ and 2.5 U TaKaRa LA Taq (Takara Bio Inc., Kyoto, Japan) was prepared in 20 μL of a reaction solution. We conducted 35 cycles of PCR, comprising a 30 s denaturing phase at 94 ℃, a 30 s annealing phase at 50 ℃, and a synthesis phase for 4 min at 72 ℃, was conducted. The PCR product was run on a 1% agarose gel, and was detected. A 1.4 kp RT-PCR product was purified by PCR SURPEC (Takara Bio Inc., Kyoto, Japan). Eight microlitres of purified PCR solution was gently mixed with a ligation solution containing 50 ng pGEM T Easy vector (Promega, Madison, WI), 1× Rapid ligation buffer, and 3 units of T4 DNA ligase. Ligation was carried out at 4 °C overnight. The recombinant DNA ligated with vector pGEM T Easy Vector was transformed into *E. coli* JM109. Plasmid DNA was isolated by alkaline lysis method (Birnboim and Doly, 1979) and the nucleotide sequence of the insert DNA was subjected to cycle sequence reaction using BigDye™ Terminator v3.1 Cycle Sequencing Kit (Thermo Fisher Scientific, San Jose, CA) with T7 universal primer and SP6m universal primer in the vectors, and nucleotide sequences were decoded by Thermo Fisher ABI Prism Applied Biosystems 3100 Genetic Analyzer.

**Table 1 -:** Primer sets designed for cloning cDNA and genome DNA of wheat *TaNHX* genes.

Forward primer	Reverse primer
cDNA cloning	
Na/H-F	Na/H-R
5’-GGAGAATCGCTGGGTCAATGAGTCC-3’	5’-TCAGCGCGTCGTCGAACTTGCGC-3’
CapFishing^TM^ adapter primer	
5’-AAGACGAGCACGTGCGAGCTCTTCCCC-3’	
*TaNHXa* cDNA 5’ primer	*TaNHXa* cDNA 3’ primer
5’-CCGGATGCTCATCACCAAGCCGACCC-3’	5’-GTCTACCAGGCATTCGCTTCAT-3’
*TaNHXb* cDNA 5’ primer	*TaNHXb* cDNA 3’ primer
5’-ATGCTGTGAGCATACGCCGCCCGACGAG-3’	5’-GGACGCGAAGAGGATGACCGTGCCGGCG-3’
*TaNHXc* cDNA 5’ primer	*TaNHXc* cDNA 3’ primer
5’-CTTCGATGTAGGCCAGCTTACCCCCC -3’	5’-CTTTGGTGCAGATCAGAATGACACCACC-3’
gDNA cloning	
5’ end region of*TaNHXa*	
5’-ATGGGGTACCAAGTGGTGGCGGCGCAGC-3’	5’-AAGACGAGCACGTGCGAGCTCTTCCCC-3’
middle region of *TaNHXa*	
5’-ATCACCGCCCTCATCATCGGGCTGTGC-3’	5’-CAGTAGTAGTGGATGGTGTGGGTCGGC-3’
3’ end region of*TaNHXa*	
5’-CCGGATGCTCATCACCAAGCCGACCC-3’	5’-GGACTGGAAGCAAAGTCTTATTCCTCCATC-3’
5’ end region of *TaNHXb*	
5’-CTCGTCTCGTCTCTTGTCGTGCAACACAAG-3’	5’-GGACGCGAAGAGGATGACCGTGCCGGCG-3’
middle region 1 of *TaNHXb*	
5’-ACCACCGCGCTCTTGCTTGGGCTGGGCG-3’	5’-CAGTAGTGGTGGACTGACCGTGTTGGACTC-3’
middle region 2 of *TaNHXb*	
5’-CATAGGCTCCTTGAGCTTGGAGACTACC-3’	5’-AAGGGCACTCACTTCCCTAGTGAGGTGCC-3’
3’ end region of *TaNHXb*	
5’-ATGCTGTGAGCATACGCCGCCCGACGAG-3’	5’-CGGGCGAATACTCCTTTATAATGTGCAGTG-3’
5’ end region of *TaNHXc*	
5’-CTCGCCTCGCATCCAGACTCCAGACC-3’	5’-CTTTGGTGCAGATCAGAATGACACCACC-3
middle region of *TaNHXc*	
5’-GGGATTGTTCAGCAAACTTGAGTTGGTCC-3’	5’-GCCGTAAGTGGGTCTAGGAATGACTGGC-3’
3’ end region of *TaNHXc*	
5’-CTTCGATGTAGGCCAGCTTACCCCCC-3’	5’-CCACCTGAGTTCCTTTTATACATGAATGC-3’

### Full length sequence analysis of Na^+^/H^+^ antiporter-like cDNA

Three kinds of 1,392-1,400 bp cDNA isolated by RT-PCR using primers designed from the vacuole-type Na^+^/H^+^ antiporter genes of rice and *A. subcordata* were designated as *TaNHXa*, *b*, and *c*, respectively. Next, 5’ and 3’ ends of the cDNA were obtained using the RACE method and the total structures of cDNA were determined. CapFishing^TM^ technology (Seegene, Seoul, Korea), which employs the CapFishing^TM^ adapter and the oligo(dt) primer, was used to obtain 5’ end fragments of *TaNHXa*, *b*, and *c*. The first-strand cDNAs were synthesized from CapFishing^TM^ adapter-added total RNA by SuperScript Ⅲ reverse transcriptase (Invitrogen/Life Technologies, Carlsbad, CA) using a reverse primer (5’ - AAGACGAGCACGTGCGAGCTCTTCCCC - 3’) designed from 3’ side of the 1,392 bp segment of *TaNHXa* cDNA. Then, the PCR reaction for amplifying the double strand cDNA was carried out using the 5’ CapFishing^TM^ adapter primer (5’-GTCTACCAGGCATTCGCTTCAT-3’) and the 3’ *TaNHXa* primer (5’-AAGACGAGCACGTGCGAGCTCTTCCCC-3’) in a 30 μl mixture including 4 μl of the first strand cDNA solution, 0.5 μM of each primer, 1 mM dNTPs, 1× GC buffer Ⅰ and 2.5 U of TaKaLa LA Taq polymerase. The cycling profile was: 35 cycles of 94 ˚C for 30 s, 60 ˚C for 30 s, 72 ˚C for 1 min. In the same way as for cloning 5’ end sequence of *TaNHXb* and *c*, a reverse primers were designed from 3’ side of the 1,400 bp *TaNHXb* cDNA segment (5’-GGACGCGAAGAGGATGACCGTGCCGGCG-3’) and from 3’ side of the 1,400 bp *TaNHXc* cDNA (5’-CTTTGGTGCAGATCAGAATGACACCACC-3’), respectively. PCR was conducted according to 35 cycles of 94 ˚C for 30 s, 62 ˚C for 30 s, 72 ˚C for 2 min for 5’end of *TaNHXb*, and 35 cycles of 94 ˚C for 30 sec, 64 ˚C for 30 sec, 72 ˚C for 2 min for 5’end of *TaNHXc*, respectively.

Meanwhile, to obtain the 3’ ends of *TaNHXa*, *b*, and *c*, the first-strand cDNAs were synthesized from CapFishing^TM^ adapter-added total RNA by SuperScript Ⅲ reverse transcriptase using an oligo(dt) primer. Then, PCR reaction for amplifying the double strand cDNA was carried out using the a forward primers designed from the 5’ side of the 1,392-1,400 bp cDNA segment of *TaNHXa*, *b*, and *c* (*TaNHXa*: 5’-CCGGATGCTCATCACCAAGCCGACCC-3’, *TaNHXb*: 5’-ATGCTGTGAGCATACGCCGCCCGACGAG-3’, *TaNHXc*: 5’- CTTCGATGTAGGCCAGCTTACCCCCC-3’) and oligo(dt) primer. PCR was conducted according to 35 cycles of 94 ˚C for 30 s, 61 ˚C for 30 s, 72 ˚C for 4 min for 3’end of *TaNHXa*, and 35 cycles of 94 ˚C for 30 s, 63 ˚C for 30 s, 72 ˚C for 2 min for 5’end of *TaNHXb*, and 35 cycles of 94 ˚C for 30 s, 57 ˚C for 30 s, 72 ˚C for 2 min for 5’end of *TaNHXc* respectively. The double-strand cDNA products were purified by PCR SURPEC, TA-cloned by pGEM T Easy Vector, and transformed into *E. coli* JM109. The recombinant plasmids were isolated by alkaline lysis method and subjected to cycle sequencing reaction with T7 universal primer and SP6m universal primer, and nucleotide sequences were decoded by using Thermo Fisher ABI Prism Applied Biosystems 3100 Genetic Analyzer. The entire nucleotide sequence of the cDNA was then determined by assembling overlapped read sequences. Nucleotide sequences were compared with sequences in the nonredundant GenBank+EMBL+DDBJ databases with BLASTN homology search software (Altschul et al., 1997). A phylogenetic tree was constructed with Phylogenetic analysis pipeline by ETE3 (Huerta-Cepas et al., 2016).

### Full length sequence analysis of genome DNA of Na^+^/H^+^ antiporter-like genes

Using primers based on the total nucleotide sequence of cDNA of *TaNHXa*, *b*, and *c*, genomic DNA fragments containing introns of these genes were amplified by PCR, and the entire structure of the *TaNHX* genes was determined. By designing primers from the 5’ side, the central region, and the 3’ side of the gene DNA were amplified, we conducted PCR using the genomic DNA of Chinese Spring, a bread wheat variety. Genome DNA was extracted by using the cetyl trimethylammonium bromide (CTAB) method (Murray and Thompson, 1980) and was then used as a genome DNA template. As for cloning 5’ region of *TaNHXa* genomic sequence, a forward primer (5’-ATGGGGTACCAAGTGGTGGCGGCGCAGC-3’) and a reverse primer (5’-AAGACGAGCACGTGCGAGCTCTTCCCC-3’) were designed and PCR was conducted according to 35 cycles of 94 ˚C for 30 s, 60 ˚C for 30 s, 72 ˚C for 5 min. As for cloning middle region of *TaNHXa* genomic sequence, a forward primer (5’-ATCACCGCCCTCATCATCGGGCTGTGC-3’) and a reverse primer (5’-CAGTAGTAGTGGATGGTGTGGGTCGGC-3’) were designed and PCR was conducted according to 35 cycles of 94 ˚C for 30 s, 60 ˚C for 30 s, 72 ˚C for 5 min. As for cloning 3’ region of *TaNHXa* genomic sequence, a forward primer (5’-CCGGATGCTCATCACCAAGCCGACCC-3’) and a reverse primer (5’-GGACTGGAAGCAAAGTCTTATTCCTCCATC-3’) were designed and PCR was conducted according to 35 cycles of 94 ˚C for 30 s, 63 ˚C for 30 s, 72 ˚C for 5 min. As for cloning 5’ region of *TaNHXb* genomic sequence, a forward primer (5’-CTCGTCTCGTCTCTTGTCGTGCAACACAAG-3’) and a reverse primer (5’-GGACGCGAAGAGGATGACCGTGCCGGCG-3’) were designed and PCR was conducted according to 35 cycles of 94 ˚C for 30 s, 62 ˚C for 30 s, 72 ˚C for 5 min. As for cloning middle region of *TaNHXb* genomic sequence, a forward primer (5’-ACCACCGCGCTCTTGCTTGGGCTGGGCG-3’) and a reverse primer (5’-CAGTAGTGGTGGACTGACCGTGTTGGACTC-3’) were designed and PCR was conducted according to 35 cycles of 94 ˚C for 30 s, 65 ˚C for 30 s, 72 ˚C for 7 min. As for cloning second middle region of *TaNHXb* genomic sequence, a forward primer (5’-CATAGGCTCCTTGAGCTTGGAGACTACC-3’) and a reverse primer (5’-AAGGGCACTCACTTCCCTAGTGAGGTGCC-3’) were designed and PCR was conducted according to 35 cycles of 94 ˚C for 30 s, 60 ˚C for 30 s, 72 ˚C for 5 min. As for cloning 3’ region of *TaNHXb* genomic sequence, a forward primer (5’-ATGCTGTGAGCATACGCCGCCCGACGAG-3’) and a reverse primer (5’-CGGGCGAATACTCCTTTATAATGTGCAGTG-3’) were designed and PCR was conducted according to 35 cycles of 94 ˚C for 30 s, 63 ˚C for 30 s, 72 ˚C for 5 min. As for cloning 5’ region of *TaNHXc* genomic sequence, a forward primer (5’-CTCGCCTCGCATCCAGACTCCAGACC-3’) and a reverse primer (5’-CTTTGGTGCAGATCAGAATGACACCACC-3’) were designed and PCR was conducted according to 35 cycles of 94 ˚C for 30 s, 60 ˚C for 30 s, 72 ˚C for 5 min. As for cloning middle region of *TaNHXc* genomic sequence, a forward primer (5’-GGGATTGTTCAGCAAACTTGAGTTGGTCC-3’) and a reverse primer (5’-GCCGTAAGTGGGTCTAGGAATGACTGGC-3’) were designed and PCR was conducted according to 35 cycles of 94 ˚C for 30 s, 60 ˚C for 30 s, 72 ˚C for 5 min. As for cloning 3’ region of *TaNHXc* genomic sequence, a forward primer (5’-CTTCGATGTAGGCCAGCTTACCCCCC-3’) and a reverse primer (5’-CCACCTGAGTTCCTTTTATACATGAATGC-3’) were designed and PCR was conducted according to 35 cycles of 94 ˚C for 30 s, 57 ˚C for 30 s, 72 ˚C for 5 min. Each PCR reaction mixture consisted of 920 ng of Chinese Spring genomic DNA, 0.25 μM of each primer, 1 mM dNTPs, 1× GC buffer Ⅰ, and 2.5 U of TaKaLa LA Taq polymerase in a 60 μl solution. The amplified genomic DNA products were purified by PCR SURPEC, TA-cloned by pGEM T Easy Vector, and transformed into *E. coli* JM109. The recombinant plasmids were isolated by alkaline lysis method and sequenced by cycle reaction with T7 universal primer and SP6m universal primer. Nucleotide sequences of these deletion clones were decoded by using Thermo Fisher ABI Prism Applied Biosystems 3100 Genetic Analyzer. The entire nucleotide sequences of the *TaNHX* genes were then determined by assembling overlapped read sequences. Nucleotide sequences were compared with sequences in the nonredundant GenBank+EMBL+DDBJ databases with BLASTN homology search software (Altschul et al., 1997). A phylogenetic tree was constructed with Phylogenetic analysis pipeline by ETE3 (Huerta-Cepas et al., 2016).

### Chromosome localization

To determine the chromosome location of *TaNHXb* gene, specific primers were designed from 706 bp of intron 2 sequence, and PCR was conducted using genomic DNA from a series of ditelosomics of Chinese Spring as templates. As for diagnosis of *TaNHXb*, a forward primer (5’-GTATGTTCTTCTTATGCAAATTAC-3’) and a reverse primer (5’-CTGATCAGGGATAGGGGAAAAGAG-3’) were designed and PCR was conducted according to 35 cycles of 94 ˚C for 30 s, 57 ˚C for 30 s, 72 ˚C for 1 min. PCR reaction mixture consisted of 920 ng of CS genomic DNA, 0.25 μM of each primer, 1 mM dNTPs, 1× GC buffer Ⅰ, and 2.5 U of TaKaLa LA Taq polymerase in a 60 μl solution.

## Results

### Three types of Na^+^/H^+^ antiporter-like cDNA of bread wheat

**Figure 1 -:**
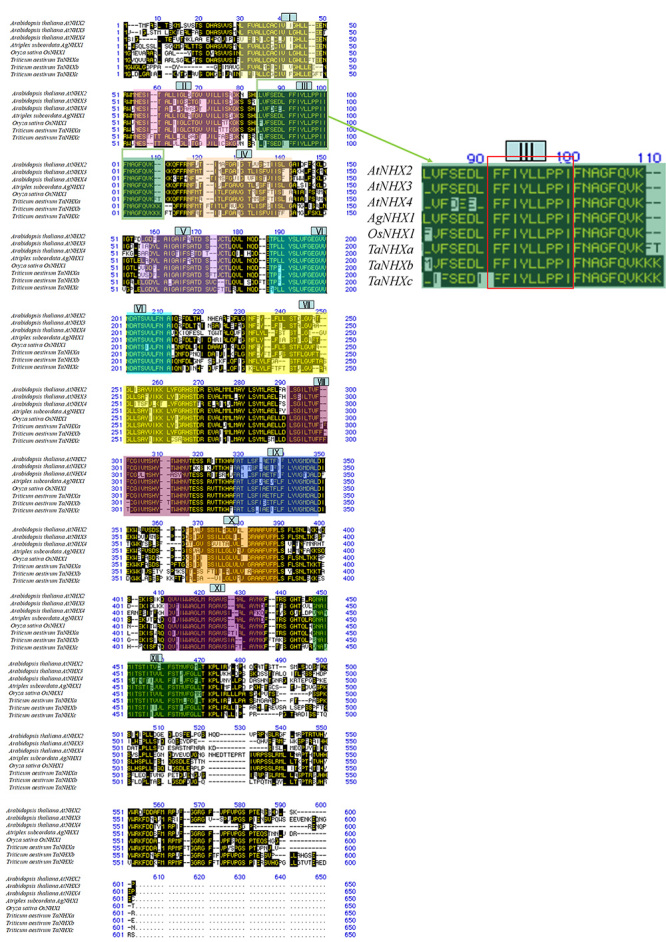
Deduced amino acid sequences of wheat Na^+^/H^+^ antiporter-like transcripts. To conduct cloning of Na^+^/H^+^ antiporter genes expressed in bread wheat, primers were designed by finding similar common regions in *OsNHX1* of rice and *AgNHX1* of *Atriplex subcordata*. After spraying young plants of the bread wheat variety Chinese Spring with 0.5 M NaCl, RNA was extracted. Three kinds of 1,392~1,400 bp cDNA were isolated by RT-PCR using primers designed from the cell vacuole-type Na^+^/H^+^ antiporter genes of rice and *Atriplex subcordata*. In addition, tip ends of the cDNA were obtained with the RACE method and the total structures determined by using primers based on the nucleotide sequence of cDNA clones of *TaNHXa*, *b*, and *c*. Three vacuolar-type antiporter-like genes, *TaNHXa*, *b*, and *c*, encoding 539~547 sequences of amino acid residues with 12 transmembrane domains containing amyloid binding sites, a characteristic of the vacuolar-type Na^+^/H^+^ antiporter, were isolated from bread wheat. The total lengths of *TaNHXa*, *b* and *c* were 2,414 bp, 539 a.a. (GenBank accession number, MT550001), 1,898 bp, 539 a.a. (MT550002), and 1,928 bp, 547 a.a. (MT550003), respectively. Comparison of the amino acid sequences of Na^+^/H^+^ antiporter genes from *Arabidopsis thaliana*, *Atriplex subcordata* and rice and those from the 3 Na^+^/H^+^ antiporter genes of bread wheat cloned in this study revealed 3 *TaNHX* genes with 12 transmembrane domains allowing them to bind to the cell vacuole membrane, a characteristic of Na^+^/H^+^ antiporter genes. The shaded areas represent transmembrane domains and Roman numerals refer to domain numbers. In the third transmembrane domain of all *TaNHX*, amyloid binding sites (FFIYLLPP), which are also characteristic of Na^+^/H^+^ antiporter genes, were observed.

Three vacuolar-type antiporter-like genes, *TaNHXa*, *b* and *c*, encoding 556-564 residues of amino acids with 12 transmembrane domains containing amyloid binding sites, characteristic of the vacuolar-type Na^+^/H^+^ antiporter, were isolated from bread wheat (Figure 1). The total lengths of *TaNHXa*, *b,* and *c* were 2,414 bp, 539 a.a. (GenBank accession number, MT550001); 1,898 bp, 539 a.a. (MT550002); and 1,928 bp, 547 a.a. (MT550003); respectively. Comparison of the amino acid sequences of Na^+^/H^+^ antiporter genes from *A. thaliana*, *A. subcordata*, and rice and those from the three Na^+^/H^+^ antiporter genes of bread wheat cloned in this study revealed three *TaNHX* genes encoding proteins with 12 transmembrane domains allowing them to bind to the cell vacuole membrane, which is a characteristic of Na^+^/H^+^ antiporter genes. The shaded areas represent transmembrane domains and numerical figures refer to domain numbers (Figure 1). In the third transmembrane domain of all *TaNHXs*, amyloid binding sites (FFIYLLPP), which are also characteristic of Na^+^/H^+^ antiporter genes, were observed.

**Figure 2 -:**
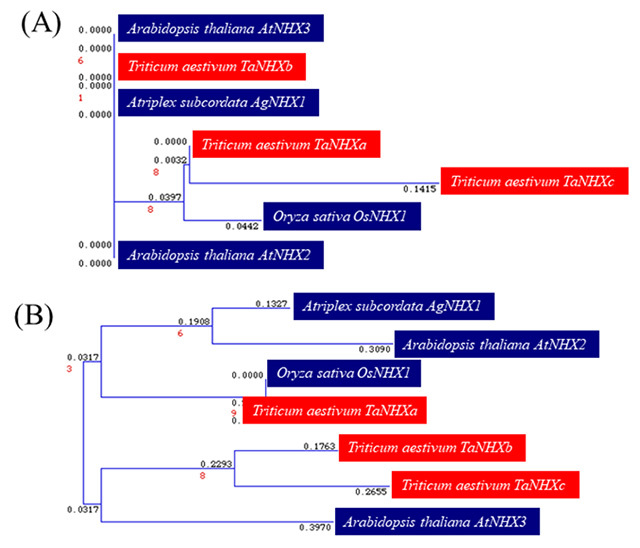
Evolutionary tree of the 12 transmembrane domains that are characteristics of Na^+^/H^+^ antiporter genes and among the regions between the transmembrane domains, (A) high homology in transmembrane domains among the species, (B) diversity in the regions between the transmembrane domains. When an evolutionary tree was created using the NJ method for the region between the 12 transmembrane domains that are characteristics of Na^+^/H^+^ antiporter genes and transmembrane domains, the amino acid sequences of these regions were in good agreement between bread wheat (*TaNHXa*, *TaNHXb*, *TaNHXc*), rice (*OsNHX1*), Atriplex subcordata (*AgNHX*) and Arabidopsis thaliana (*AtNHX2*, *AtNHX3*, *AtNHX4*). Moreover, the amyloid binding sites were completely in agreement in all plants. Considerable differences were observed depending on the plant species in the regions between the transmembrane domains. However, *TaNHXa* of bread wheat showed a high degree of homogeneity with *OsNHX1*of rice between domains and a close relationship on the whole.

When an evolutionary tree was created using the NJ method for the 12 transmembrane domains that are characteristic of Na^+^/H^+^ antiporter genes and for the regions between the transmembrane domains (Figure 2), the amino acid sequences of these regions were in good agreement between bread wheat (*TaNHXa, TaNHXb,* and *TaNHXc*), rice (*OsNHX1*), *A. subcordata* (*AgNHX*) and *A. thaliana* (*AtNHX2, AtNHX3,* and *AtNHX4*). Moreover, the amyloid binding sites were completely in agreement in all plants. Considerable differences were observed depending on the plant species in the regions between the transmembrane domains. However, *TaNHX1* of bread wheat showed a high degree of homogeneity with *OsNHX1* of rice between domains and a close relationship on the whole.

**Figure 3 -:**
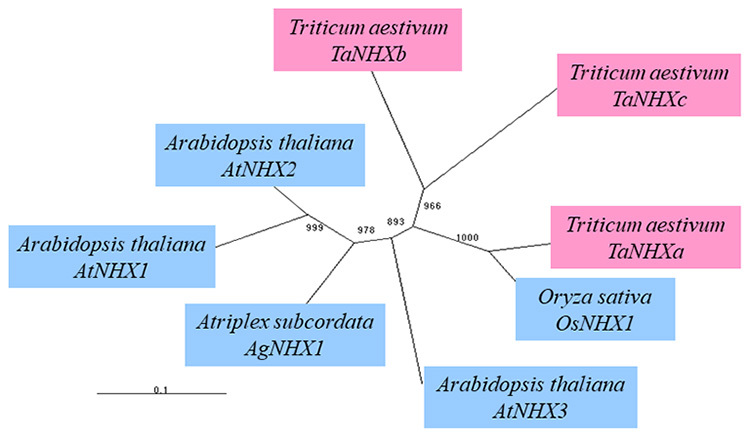
Evolutionary tree of deduced amino acid sequences of wheat Na^+^/H^+^ antiporter-like genes. Homogeneity was examined by creating an evolutionary tree of all amino acid sequences of the *NHX* genes. *TaNHXa* of bread wheat showed the highest homogeneity with *OsNHX1* of rice, while *TaNHXb* and *TaNHXc* were hereditarily different from the Na^+^/H^+^ antiporter genes of the other plants, making them independent. *TaNHXa* showed the highest homogeneity of 89% with *OsNHX1* of rice at the amino acid sequence level, indicating a close relationship. *TaNHXa* showed 72 % homogeneity with *AgNHX1* of *Atriplex subcordata* and 62 % with *AtNHX1* of *Arabidopsis thaliana*. On the other hand, *TaNHXb* showed homogeneity of about 60 % with *TaNHXa*, *OsNHX1*, *AgNHX1* and *AtNHX1*. Moreover, the percentage homogeneity of *TaNHXc* with *TaNHXa* and *TaNHXb* was around 50%, and although the percentage homogeneity with *OsNHX1*, *AgNHX1* and *AtNHX1* was low (46 to 63 %), the 124~149th amino acid residues were similar to *AgNHX1* of *Atriplex subcordata*. It is assumed that of the 3 kinds of Na^+^/H^+^ antiporter genes in bread wheat, *TaNHXa* is of the same evolutionary origin as *OsNHX1* of *Oryza sativa*. The two other genes, *TaNHXb* and *TaNHXc*, showed low homogeneity with the other plants, possibly contributing to the adaptability of bread wheat to semi-dry areas. Although only 1 kind of Na^+^/H^+^ antiporter gene was obtained from rice and *Atriplex subcordata*, 3 were obtained from bread wheat. Since bread wheat is a heteromerous hexaploid with 3 kinds of genome, this suggests that each genome independently has one gene.

Homogeneity was examined by creating an evolutionary tree of all amino acid sequences encoded by the *NHX* gene (Figure 3). *TaNHXa* of bread wheat showed the highest homogeneity of rice *OsNHX1*, while *TaNHXb* and *TaNHXc* were hereditarily different from the Na^+^/H^+^ antiporter genes of the other plants, making them independent. *TaNHXa* showed the highest homogeneity of 89% with *OsNHX1* of rice at the amino acid sequence level, indicating a close relationship*. TaNHXa* showed 72% homogeneity with *AgNHX1* of *A. subcordata* and 62% with *AtNHX1* of *A. thaliana*. However, *TaNHXb* showed homogeneity of approximately 60% with *TaNHXa*, *OsNHX1*, *AgNHX1,* and *AtNHX1*, respectively. Moreover, the percentage homogeneity of *TaNHXc* with *TaNHXa* and *TaNHXb* was approximately 50%, and although the percentage homogeneity of *OsNHX1*, *AgNHX1* and *AtNHX1* was low (46%-63%), the 124-149th amino acid residues were similar to *AgNHX1* of *A. subcordata*. It is assumed that of the three types of Na^+^/H^+^ antiporter genes in bread wheat, *TaNHXa* is of the same evolutionary origin as *OsNHX1* of *Oryza sativa*. The two other genes, *TaNHXb* and *TaNHXc*, showed low homogeneity with the other plants, possibly contributing to the adaptability of bread wheat to semidry areas. Although only one kind of Na^+^/H^+^ antiporter gene was obtained from rice and *A. subcordata*, respectively, three were obtained from bread wheat. Since bread wheat is a heteromerous hexaploid with three genomes, this suggests that each genome independently contains one of the genes.

### Genomic structure of Na^+^/H^+^ antiporter-like genes of bread wheat

**Figure 4 -:**
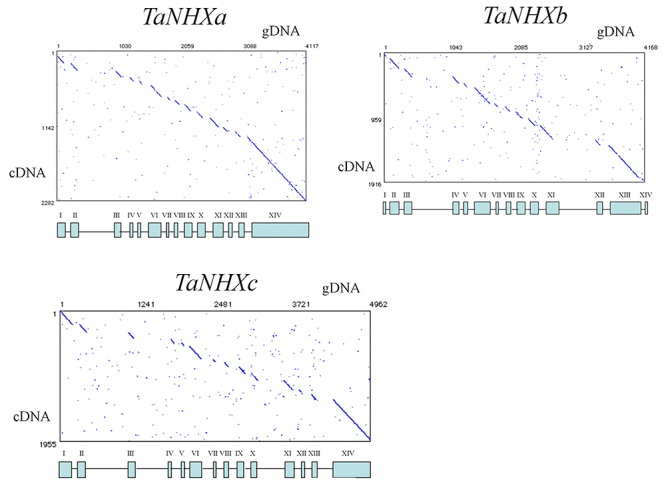
Exon/intron structure of Na^+^/H^+^ antiporter-like genes of bread wheat revealed by comparison of genomic DNA and cDNA. The nucleotide sequences of genomic DNA fragments amplified by PCR were determined. For the *TaNHXa* gene, nucleotide sequences of 4,255 bp (GenBank accession number, MT549998) in total length were determined by obtaining DNA fragments of 1.9 kb from the central region, of 290 bp and 1.3 kb from the 5' tip end, and of 1.1 kb from the 3' tip end. For the *TaNHXb* gene, nucleotide sequences of 4,167 bp (MT549999) bp in total length were determined by obtaining DNA fragments of 2.2 kb from the central region, of 370 bp and 1,410 bp from the 5' tip end, and of 630 bp and 430 bp from the 3' tip end. For the *TaNHXc* gene, nucleotide sequences of 4,962 bp (MT550000) in total length were determined by obtaining DNA fragments of 944 bp from the 5' tip end, 2.7 kb from the central region and 570 bp from the 3' tip end. The exon/intron structures of the *TaNHX* genes were determined by comparing cDNA and genomic DNA. This figure shows exon/intron structures clarified by homologous regions of genomic DNA of *TaNHXa*, *b*, and *c* with their cDNA. Each *TaNHX* consisted with 14 exons and 13 introns. Exon sizes ranged from a maximum of 489 bp to a minimum of 47 bp, while introns ranged from a maximum of 653 bp to a minimum of 34 bp in *TaNHXb*. Exon sizes ranged from a maximum of 605 bp to a minimum of 47 bp, while intron sizes ranged from a maximum of 462 bp to a minimum of 77 bp in *TaNHXc*.

**Figure 5 -:**
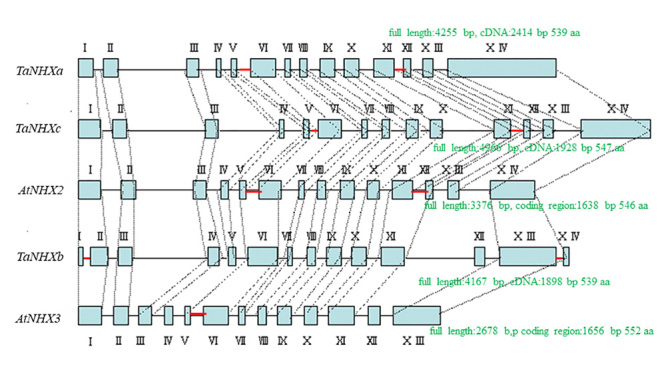
Structural relationship between the wheat Na^+^/H^+^ antiporter-like genes and *Arabidopsis thaliana* antiporter genes. The total DNA structures of *TaNHXa*, *b* and *c* were compared with those of *AtNHX2* and *3* of *Arabidopsis thaliana*. *TaNHXa*, *b* and *c* were 22~55 % longer than *AtNHX2*, and *3* in total length, 15%~17% longer in the exon, and 28%~122% longer in the intron. This reflects the enlargement of the genome size in bread wheat. *TaNHXa*, *b*, and *c* have 13 intron sequences; that is, one more intron than *AtNHX3*. Moreover, while the nucleotide sequences of the exons showed 64%~78% homogeneity, those of the introns were as low as 54%~58%, and significant mutations were observed compared with the exons in both length and sequence. Although the positions of introns lying in the coding regions of *TaNHX* and *AtNHX* did not differ remarkably, the region corresponding to the 5th intron of *TaNHXa, c, AtNHX2* and *3* did not exist in *TaNHXb*, and in the addition the 1st and 13th introns were newly developed. Moreover, the region corresponding to the 11th intron in *TaNHXa, c* and *AtNHX2* did not exist in *TaNHXb* and *AtNHX3*.

**Figure 6 -:**
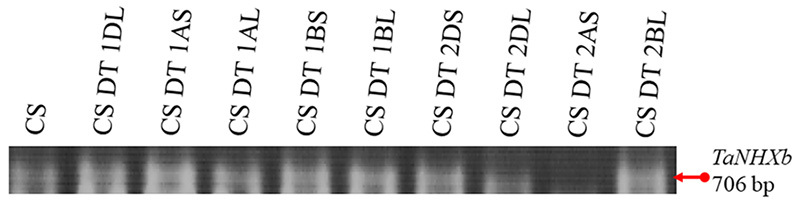
Chromosomal localization of the wheat Na^+^/H^+^ antiporter gene by using ditelosomic series. To determine the chromosome location of *TaNHXb* gene, specific primers were designed from 706 bp of intron 2 sequence, and PCR was conducted using genomic DNA from a series of ditelosomics of Chinese Spring as templates. The 706 bp band of *TaNHXb* was not found in a ditelosomic line lacking the short arm of chromosome 2A. Therefore, *TaNHXb* was localized on chromosome 2A. DT: ditelosomic.

The nucleotide sequences of genomic DNA fragments amplified by PCR were determined. For the *TaNHXa* gene, nucleotide sequences of 4,255 bp (GenBank accession number MT549998) in total length were determined by obtaining DNA fragments of 1.9 kb from the central region, of 290 bp and 1.3 kb from the 5’ tip end, and of 1.1 kb from the 3’ tip end. For the *TaNHXb* gene, nucleotide sequences of 4,167 bp (MT549999) in total length were determined by obtaining DNA fragments of 2.2 kb from the central region, of 370 bp and 1,410 bp from the 5’ tip end, and of 630 bp and 430 bp from the 3’ tip end. For the *TaNHXc* gene, nucleotide sequences of 4,966 bp (MT550000) in total length were determined by obtaining DNA fragments of 944 bp from the 5’ tip end, 2.7 kb from the central region and 570 bp from the 3’ tip end. The exon/intron structures of the *TaNHX* genes were determined by comparing cDNA and genomic DNA. Figure 4 shows the homologous regions of genomic DNA of *TaNHXa, b* and *c* together with exon/intron structures. Each *TaNHX* consisted with 14 exons and 13 introns (Figure 5). Exon sizes ranged from a maximum of 489 bp to a minimum of 47 bp, while introns ranged from a maximum of 653 bp to a minimum of 34 bp in *TaNHXb*. Exon sizes ranged from a maximum of 605 bp to a minimum of 47 bp, while intron sizes ranged from a maximum of 462 bp to a minimum of 77 bp in *TaNHXc* (Figure 5). To determine the chromosome location of *TaNHXb* gene, specific primers were designed from 706 bp of intron 2 sequence, and PCR was conducted using genomic DNA from a series of ditelosomics of Chinese Spring as templates. The 706 bp band of *TaNHXb* was not found in a ditelosomic line lacking the short arm of chromosome 2A. Therefore, *TaNHXb* was localized on chromosome 2A (Figure 6).

## Discussion

The total DNA structures of *TaNHXa, b,* and *c* clarified in this study were compared with the genomic DNA structures of *AtNHX2* and *3* of *Arabidopsis thaliana*. *TaNHXa, b,* and *c* were 22~55 % longer than those of *AtNHX2,* and *3* in total length, 15%~17% longer in the exon, and 28%~122% longer in the intron (Figure 5). This reflects the enlargement of the genome size in bread wheat. *TaNHXa, b* and *c* have 13 intron sequences; that is, one more intron than *AtNHX3*. Moreover, while the nucleotide sequences of the exons showed 64%-78% homogeneity, homogeneity in the introns was as low as 54%-58% and significant mutations were observed compared with the exons in both length and sequence. Although the positions of introns lying in the coding regions of the *TaNHX* and *AtNHX* genes did not differ remarkably, the region corresponding to the 5th intron of *TaNHXa, c*, *AtNHX2* and *3* did not exist in *TaNHXb*, other than in the newly developing 1st and 13th introns. Moreover, structural mutations were found whereby the region corresponding to the 11th intron in *TaNHXa, c* and *AtNHX2* did not exist in *TaNHXb* and *AtNHX3*. cDNA of *TaNHXa* showed 99.91% homology to *TaNHX2* (AY040246: 2422 bp, 538 aa). *TaNHX2* was cloned by screening a salt-stressed wheat cDNA library (Brini et al., 2005). Expressed *TaNHX2* protein suppressed the salt sensitivity of a yeast mutant strain by increasing its K^+^ content when exposed to salt stress (Xu et al., 2013). *TaNHX2* also increased the tolerance of the strain to potassium stress. Transgenic eggplants (*Solanum melongena* L.) by introducing *TaNHX2* gene in to the eggplant genome via *Agrobacterium*-mediated transformation to confer salinity tolerance (Yarra et al., 2019). The overexpression of *TaNHX2* gene in transgenic sunflower (*Helianthus annuus* L) conferred improved salinity stress tolerance, and growth performance (Mushke et al., 2019). *TaNHX2* transgenic alfalfa (*Medicago sativa* L.) accumulated more K^+^ and less Na^+^ in leaves than did the wild-type plants (Zhang et al., 2015). In this study, we firstly revealed the whole genomic structure of one of the *TaNHX2* alleles (*TaNHXa*, MT549998: gDNA 4,255 bp; MT550001: cDNA 2,414 bp, 539 a.a.), which consisted of 14 exons and 13 introns. cDNA of *TaNHXb* showed 96.55% homology to *Aegilops tauschii* sodium/hydrogen exchanger 2-like cDNA (XM020336845: 2172 bp). cDNA of *TaNHXc* showed 98.51% homology to *Thinopyrum elongatum* Na^+^/H^+^ antiporter NHX1 cDNA (AF507044: 1955 bp). In both cases, we firstly revealed whole genomic structure of new genes of *Triticum aestivum*, *TaNHXb* (MT549999: gDNA 4,167 bp; MT550002: cDNA 1,898 bp, 539 a.a.) *and TaNHXc* (*TaNHXb*, MT550000: genome DNA 4,966 bp; MT550003: cDNA 1,928 bp, 547 a.a.).

**Figure 7 -:**
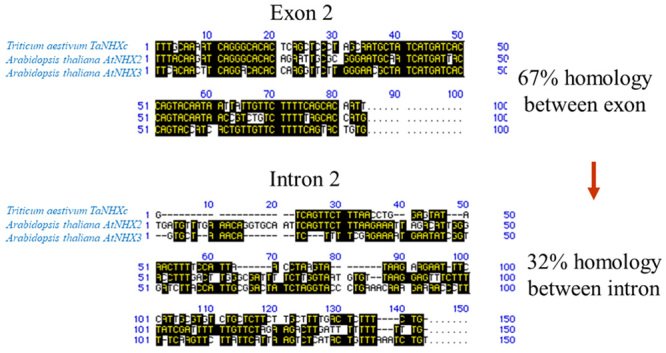
Conserved exons and diverged introns of the Na^+^/H^+^ anti porter gene between bread wheat and *Arabidopsis thaliana*. When homogeneity was analyzed regarding the 2nd exons and 2nd introns adjoining each other, homogeneity of the former was shown to be 67% among the 3 genes, while that of the latter was 32%. Introns showed a significant divergence between bread wheat and *Arabidopsis thaliana* compared with exons. Moreover, when the exon and intron structures of adjoining *TaNHXa*, *b*, and *c* were compared, the exons showed 64%~78% homogeneity among the 3 genes, while the homogeneity of introns was as low as 54%~56%, and there was a significant mutation in the intron. The level of homogeneity was high in the order of the transmembrane domain, regions between domains (of exons) and introns.

When the homogeneity of the second exon and second intron adjoining each other was analyzed, the homogeneity of the former was shown to be 67% among the three genes, while that of the latter was 32% (Figure 7). Introns showed significant divergence between bread wheat and *A. thaliana* compared with exons. Moreover, when the exon and intron structures of the adjoining *TaNHXa*, *b*, and *c* were compared, the exons showed 64%-78% homogeneity among the three genes, while the homogeneity of introns was as low as 54%-56% and there was a significant mutation in the intron. The level of homogeneity was higher in the following order, namely, the transmembrane domain, exon regions between domains and introns. When an evolutionary tree was created for the corresponding introns, no relationship was observed, with most introns largely differentiating. There was, however, a weak relationship between the 1st intron of *TaNHXa*, *c, AtNHX2*, and *3* and the 2nd intron of *TaNHXb* and between the 9th intron of *TaNHXb*, *c*, *AtNHX2*, and *3* and the 8th intron of *TaNHXa*, indicating two origins for Na^+^/H^+^ antiporter genes. As shown above, bread wheat expresses different types of cell vacuole-type sodium/proton antiporter genes.

## References

[B1] Al-Harrasi I, Jana GA, Patankar HV, Al-Yahyai R, Rajappa S, Kumar PP, Yaish MW (2020). A novel tonoplast Na+/H+ antiporter gene from date palm (PdNHX6) confers enhanced salt tolerance response in Arabidopsis. Plant Cell Rep.

[B2] Altschul SF, Madden TL, Schaffer AA, Zhang J, Zhang Z, Miller W, Lipman D (1997). Gapped BLAST and PSI-BLAST: a new generation of protein database search programs. Nucleic Acids Res.

[B3] Apse MP, Aharon GS, Snedden WA, Blumwald E (1999). Salt tolerance conferred by overexpression of a vacuolar Na+/H+ antiport in Arabidopsis. Science.

[B4] Ayadi M, Martins V, Ayed BR, Jbir R, Feki M, Mzid R, Géros H, Aifa S, Hanana M (2020). Genome wide identification, molecular characterization, and gene expression analyses of grapevine NHX antiporters suggest their involvement in growth, ripening, seed dormancy, and stress response. Biochem Genet.

[B5] Birnboim HC, Doly J (1979). A rapid alkaline extraction procedure for screening recombinant plasmid DNA. Nucleic Acids Res.

[B6] Brini F, Gaxiola RA, Berkowitz GA, Masmoudi K (2005). Cloning and characterization of a wheat vacuolar cation/proton antiporter and pyrophosphatase proton pump. Plant Physiol and Bioch.

[B7] Chanroj S, Wang G, Venema K, Zhang MW, Delwiche CF, Sze H (2012). Conserved and diversified gene families of monovalent cation/H+ antiporters from algae to flowering plants. Front Plant Sci.

[B8] Chomczynski P, Sacchi N (2006). Single-step method of RNA isolation by acid guanidinium thiocyanate-phenol-chloroform extraction: twenty-something years on. Nat Protoc.

[B9] Counillon L, Pouyssegur J (2000). The Expanding Family of Eucaryotic Na+/H+ Exchangers. J Biol Chem.

[B10] Fukuda A, Nakamura A, Tanaka Y (1999). Molecular cloning and expression of the Na+/H+ exchanger gene in Oryza sativa. Biochim Biophys Acta.

[B11] Gaxiola RA, Rao R, Sherman A, Grisafi P, Alper SL, Fink GR (1999). The Arabidopsis thaliana proton transporters, AtNhx1 and Avp1, can function in cation detoxification in yeast. Genetics.

[B12] Hamada A, Shono M, Xia T, Ohta M, Hayashi Y, Tanaka A, Hayakawa T (2001). Isolation and characterization of a Na+/H+ antiporter gene from the halophyte Atriplex gmelini. Plant Mol Biol.

[B13] Hanana M, Cagnac O, Zarrouk M, Blumwald E (2009). Biological Roles of NHX vacuolar antiport: achievements and prospects of plant breeding. Botany.

[B14] Huerta-Cepas J, Serra F, Bork P (2016). ETE 3: Reconstruction, analysis, and visualization of phylogenomic data. Mol Biol Evol.

[B15] Jia Q, Zheng C, Sun S, Amjad H, Liang K, Lin W (2018). The role of plant cation/proton antiporter gene family in salt tolerance. Biol Plantarum.

[B16] Li W, Du J, Feng H, Wu Q, Xu G, Shabala S, Yu L (2020). Function of NHX-type transporters in improving rice tolerance to aluminum stress and soil acidity. Planta.

[B17] Murray M, Thompson WF (1980). Rapid isolation of high molecular weight plant DNA. Nucleic Acids Res.

[B18] Mushke R, Yarra R, Kirti PB (2019). Improved salinity tolerance and growth performance in transgenic sunflower plants via ectopic expression of a wheat antiporter gene (TaNHX2). Mol Biol Rep.

[B19] Qiu QS (2012). Plant and Yeast NHX Antiporters: Roles in membrane trafficking. J Integr Plant Biol.

[B20] Sharma H, Taneja M, Upadhyay SK (2020). Identification, characterization and expression profiling of cation-proton antiporter superfamily in Triticum aestivum L. and functional analysis of TaNHX4-B. Genomics.

[B21] Wang ZN, Zhang JS, Guo BH, He SJ, Tian AG, Chen SY (2002). Cloning and characterization of the Na+/H+ antiport genes from Triticum aestivum. Acta Bot Sin.

[B22] Xu Y, Zhou Y, Hong S, Xia Z, Cui D, Guo J, Xu H, Jiang X (2013). Functional characterization of a wheat NHX antiporter gene TaNHX2 that encodes a K+/H+ exchanger. PLoS One.

[B23] Yarra R, Kirti PB (2019). Expressing class I wheat NHX (TaNHX2) gene in eggplant (Solanum melongena L.) improves plant performance under saline condition. Funct Integr Genomic.

[B24] Yokoi S, Francisco JQ, Beatriz C, Maria TR, Ray AB, Paul MH, Jose MP (2002). Differential expression and function of Arabidopsis thaliana NHX Na+/H+ antiporters in the salt stress response. Plant J.

[B25] Zhang HX, Hodson JN, Williams JP, Blumwald E (2001). Engineering salt-tolerant Brassica plants: Characterization of yield and seed oil quality in transgenic plants with increased vacuolar sodium accumulation. Proc Natl Acad Sci USA.

[B26] Zhang YM, Zhang HM, Liu ZH, Li HC, Guo XL, Li GL (2015). The wheat NHX antiporter gene TaNHX2 confers salt tolerance in transgenic alfalfa by increasing the retention capacity of intracellular potassium. Plant Mol Biol.

